# Aerobic Exercise and The Heart: Discussing Doses

**DOI:** 10.5935/abc.20170020

**Published:** 2017-03

**Authors:** Claudio Gil Soares de Araújo, Claudia Lucia Barros de Castro, João Felipe Franca, Christina Grüne de Souza e Silva

**Affiliations:** 1 Clínica de Medicina do Exercício, CLINIMEX, Rio de Janeiro, RJ - Brazil; 2 Universidade Federal do Rio de Janeiro (UFRJ), Rio de Janeiro, RJ - Brazil

**Keywords:** Cardiovascular Diseases, Exercise, Prevention, Running, Longevity

The recent and exciting images of the 2016 Olympic and Paralympic Games in Rio will be forever imprinted in our minds. Hundreds of world, Olympic, continental and national records were conquered with a combination of discipline, resilience, competitiveness and overcoming challenges. Overcoming them in every sense - a result of thousands of hours of training that ensure exceptional aerobic performance from athletes with powerful and extremely healthy hearts.

The theme of aerobic exercise and the heart has never been so in evidence. Thus, in the perspective of this Olympic legacy, it is very appropriate to address the relation between aerobic exercise and the heart in the Brazilian Archives of Cardiology. More specifically, it is important to discuss the most suitable posology or dosage of exercise for adults in the context of primary and secondary prevention of cardiovascular diseases. Succinctly, the objective of this viewpoint to discuss and delimit the therapeutic range of the aerobic exercise dose for the heart and make suggestions about how this dose can be individualized according to certain criteria and objectives.

## Preliminary considerations

Free animals have been and will always be active, with confinement or sedentary styles considered unnatural. Humankind, as it is known today, was able to set off from central regions and, through its own means of locomotion (exercise) spread across far areas of the planet. Seeking food and water, running from predators, from childhood to old age, have always been linked to exercise. Indeed, a sedentary lifestyle is very recent in the history of humankind.

Before approaching the theme of exercise and the heart, it is important to conceptualize some terms (Chart 1).^1,2^ Among several terms, it is worth highlighting the difference between physical activity and exercise. While both involve movement and/or muscle contraction with energy expenditure, in exercise there is movement intention (physical activity) in a structured and repetitive manner, with the objective of maintenance or optimization of physical conditioning and/or health and/or body aesthetics.^1^

**Chart 1:** Main concepts and terms relevant to the analysis of aerobic dose recommendations for the heart

Physical fitness	Ability to perform different forms of activities and physical exercise expected for their age group, gender and physical dimensions, favoring health maintenance, survival, and adequate functionally of individuals in their environment. It can be divided into aerobic and non-aerobic components (strength/muscle power, flexibility, balance, and body composition).
Physical activity	any body movement produced by skeletal muscles that results in energy expenditure.
Exercise	structured and repetitive physical activity, whose objective is the maintenance or optimization of physical conditioning and/or body aesthetics and/or health.
Sport	physical exercise of variable energy demand which involve rules and competitions.
Sedentary lifestyle	it is the condition in which there is complete absence of regular physical exercise and frequent physical activity that involves energy expenditure (> 2 to 3 times the rest value), be it of laborious nature, for transportation or leisure.
Exerciser	it is the denomination given to the individual who exercises, that is, practices physical exercise.
Athlete	those who simultaneously meet the following criteria: a) sports training to improve performance; B) actively participate in sporting competitions; c) formally registered in sporting federations; and d) having training and sporting competitions as their main interest focus or way of life.
VO_2_ max	Maximum aerobic power or maximum oxygen consumption or, simply, aerobic condition reflects the maximum quantity of oxygen that an individual can consume in one minute of an exercise activity that involves large masses or muscle groups; it can be expressed in L.min^-1^ or, ideally, relativized by body weight and expressed in mL.kg^-1^.min^-1^.
Aerobic exercise volume (h/week)	it is the number of hours (or fractions of hours) per week of aerobic exercise; it is equivalent to the product (hours/week) of the number of weekly sections by the mean duration of sessions measured in hours.
Exercise intensity	it is expressed as the caloric expenditure in relation to the rest value (1 MET); aerobic intensity (METs) is frequently expressed by adjectives - low (light); medium (moderate); high (vigorous) - according to the % of the individuals’ own VO_2_max. Low intensity is considered up to 30% of VO_2_max, medium from 30 to 60 or 70% of the VO_2_max (or anaerobic threshold); and high if it is above this value. In simple terms, it is possible to suppose that aerobic intensity is high when it is not possible to maintain normal conversation and/ or when this intensity cannot be maintained at the same level for a long period of time.
Aerobic exercise dose	it is the product of the average intensity of exercise sessions by the respective duration in hours throughout (METs-h/week) the week.

Regarding the pharmacological dose, it can generally be defined by a therapeutic range with objective minimum and maximum limits. For a given individual, it is also possible to characterize an optimal dose with the best benefit/harm ratio. In the relation between aerobic exercise and the heart, it is also possible that there is something similar; however, to better understand this matter, we will use a few extreme examples.

## From one extreme to another

In 1968, the Dallas Bed Rest Study^3^ analysed the cardiovascular effects of extreme inactivity. Five healthy young individuals were bedridden for 21 days, and at the end of this period, a reduction of 30% was observed in VO_2_max (maximum oxygen consumption), and it took them 60 days of training to recover pre-admission conditions. This 30% loss was greater then the "physiologic" reduction of aging observed in a re-evaluation done 30 years later.^4^

In October of 2016, two fantastic aerobic feats were accomplished. In the United States, Pete Kostelnick ran from San Francisco to New York (± 5,000 km), with an average of 110 km/day at different altitudes (http://www.petesfeetaa.com/).^5^ In England, Ben Smith burned over 2.5 million calories to complete 401 marathons (42.195 km) in 401 days (± 17,000 km) (http://www.the401challenge.co.uk/).^6^ These two integrate the growing contingent of ultra-runners - > 60,000 Americans in 2013 (https://www.ultrarunning.com/featured/which-state-has-the-most-ultrarunning-finishers-as-percentage-of-population/)^7^ - that is, those who participate in running competitions (most of which are off-road) over distances longer than marathons and, in general, more than 50 miles (80 km). These ultra-runners compose an advanced and differentiated group when compared to the more than half a million Americans who complete a marathon every year.

These two paragraphs above illustrate the extremes between the "almost zero" physical activity or exercise (bed rest) and running several hours on consecutive days for long periods of time, without even one day of rest. It objectively shows how exercise dosage is, in fact, very wide and more elastic than any cardiovascular drug, and it exemplifies the cardiac tolerance of certain individuals to enormous doses of aerobic exercise.

## Discussing doses: minimum, optimal, and maximum

There is a lot of epidemiological evidence that regular aerobic exercise and high functional capacity and/or aerobic condition (VO_2_max) are associated to better health and longevity, while with a sedentary lifestyle, the opposite occurs.^8^ Indeed, the VO_2_max obtained (ideally through direct measure with the analysis of expired gases) in an incremental and truly maximum exercise test is an excellent health indicator^9^ and has recently been suggested as a vital sign.^10^

Even more importantly, VO_2_max estimates the prognosis for mortality from all causes better than any other biological marker, including glycidic/lipid profile and inflammatory markers. For example, data from Myers et al.^11^ indicate that, for every 1 more MET in the aerobic condition, there is a reduction of 12% in mortality from all causes; therefore, the difference in risk between two middle-age individuals with 50% and 100% of the predicted VO_2_max (age and gender) can be up to five times, that is, respectively, something like 1% vs 5% per year. Furthermore, in an recent example from Finland, Laukkanen et al.^12^ verified that, in healthy 50-year-old men, who increased their aerobic condition (direct measure) in 1 MET after 11 years of follow-up, the risk of mortality decreased by five times, when compared to those who kept their aerobic condition, and in up to 20 times, when compared to those with VO_2_max reduced by 4 or 5 METs in the same period. Thus, having a VO_2_max equal to or above the predicted (age/gender) should become a goal in the primary and secondary prevention of cardiovascular diseases.

In the characterization of the exercise dose, it is appropriate to have a unit of measurement. While there are several units proposed in literature, a very convenient one is METs-h/week, which consists of multiplying the number of exercise hours, in a given week, by the mean intensity in METs. Thus, considering that walking fast (6 km/h) and slow running (8 km/h) demand, respectively, 4 and 8 METs, we have that: if the individual completes the dose recommended in most guidelines and walks fast for 30 minutes 5x/week (150 minutes or 2 and a half hours/week) or runs slowly for 25 minutes 3x/week (total of 75 minutes or 1.25 hours/week), the exercise dose will be the same in both examples - 2.5x4 or 1.25x8 - that is, ± 10 METs-h/week.

Two articles from 2015 brought relevant contributions regarding the relation between all-cause mortality and exercise dose, considering, separately, relative volume and intensity of the exercise. Arem et al.^13^ identified that a maximum reduction in mortality was obtained with a dose of aerobic exercise corresponding to 3 to 5x the minimum dose recommended in the guidelines, that is, something between 20 and 50 METs-h/week (running 2 to 5 hours per week at 10 km/h) and that even a very high dose of 75-100 METs-h/week (8 to 12 hours of running per week) maintained the benefits of mortality reduction. Yet Gebel et al.^14^ showed that, for a given weekly duration of exercise, benefits were greater when, in at least 30% of the duration, aerobic exercise was of high intensity. In fact, these two important facts can be conflated towards the definition of an optimum dose, and also characterize what could be denominated as extreme exerciser; that is, the one that exceeds 100 METs-h/week for long periods of time, which is still scarcely found in literature. We can then initially propose that the therapeutic range of aerobic exercise dose is between approximately 7.5 to 100 METs-h/week.

In our opinion, the issue of aerobic exercise dose should be individualized according to objectives to be reached. The only known way to increase VO_2_ max, regardless of the current level, is by incrementing the dose of aerobic exercise. Therefore, if there is a clinical objective of increasing VO_2_max to reduce future mortality rate, or for the individual's sport goal, we can define the optimum dose as the one capable of offering the desired gains within a pre-established period. On the other hand, if the objective is only to maintain an excellent VO_2_max (> 120% of the predicted for age and gender), the optimum aerobic exercise dose may be one that is able to ensure the maintenance of this privileged aerobic condition, according to cyclical adjustments recommended by the science of physical training. Chart 2 presents recommendations of aerobic dose and periodicity of re-evaluations, considering a classification founded on the % of the predicted VO_2_max obtained through assessment and based on the current exercise pattern.

**Chart 2:** Aerobic exercise dose recommendations for adults based on: predicted %VO_2_max obtained in the evaluation, and current exercise pattern

Predicted %VO2max (adjusted for age and gender) obtained in the evaluation*	Current exercise pattern	Aerobic dose	Recommendation details: practical suggestions**	Periodicity ofaerobic re-evaluations***
> 120%	regular	maintain	Evaluate according to clinical or sporting objectives.	Elective
	irregular	adjust	Make it regular (minimum of 3x/week); consider varying modality and/or dose per exercise session; intensity can be high, at least occasionally.	Biannual
100 to 120%	regular	maintain	Evaluate according to clinical and/or sporting objective.	Biannual
	irregular	adjust	Make it regular (minimum of 3x/week); increase % of high intensity in the exercise session.	Annual
80 to 99%	regular	increase	+1 day/week or > total duration or increase of mean intensity of the exercise session.	Annual
	irregular	increase	Make it regular (minimum of 4x/week); increase % high intensity in the exercise session.	Annual
	none ou almost none	start/increase	3-4x/week 20 to 40 min; increase initially duration and then mean intensity; increase mean intensity; stimulate interval training and vary aerobic modalities.	Semester
60 to 79%	regular	increase	+2 days/week or >20% total duration and increase of average intensity; mean intensity; stimulate interval training and vary aerobic modalities.	Semester
	irregular	increase	Make it regular (minimum of 4x/week); simultaneously increase volume and % high intensity.	Semester
	none ou almost none	start/increase	4 to 6x/week 15 to 45 min; increase initially duration and after reaching 150 min/week also increase mean intensity of exercise session; consider varying aerobic modalities.	Semester
< 60%	regular	increase	Make it practically daily or >30% total duration and increase mean intensity stimulate interval training and vary aerobic modalities.	Semester
	irregular	increase	Make it regular (minimum de 4x/week); significantly increase volume and % high intensity.	Semester
	none ou almost none	start/increase	4a7x/week 10 to 50 min (possible to make it 2 daily sessions of 10-15 min); increase initially duration, and after reaching 150 min/week also increase mean intensity of exercise session; consider varying aerobic modalities.	Quarterly or Semester

* preferably performed with maximum exercise test (ideally direct measure); clinical or sporting objectives can determine the need for a re-evaluation at any moment.

** if there are clinical or sporting objectives to be reached, the exercise dose should be increased according to the objective to be contemplated; on the other hand, in case of clinical restrictions, it may be convenient to reduce the dose, especially when the exercise is not performed in exercising program sessions with medical supervision.

*** the suggestion of evaluation periodicity is specific for VO2max; clinical criteria or other objectives can determine different periodicities.

## Exercise exaggeration?

In 1899, Williams & Arnold^15^ did a thorough medical evaluation of Boston Marathon runners before the competition and one hour after completion (first place time of completion = 2h54m). They concluded that participation in the marathon did not seem to cause significant damage to the cardiovascular system of those young and healthy individuals. After more than 110 years of this rich description, there is a lot of positive evidence of athletes' and exercisers' cardiac health, such as the observation that athletes who participate in longer sporting competitions^16^ and physically active adults tend to live longer,^17^ and there is also the fact that deaths are extremely rare in half-marathons and marathons, with a rate under 1/100,000 participants.^18^ In this context, it is worth mentioning the case of Alexandre Ribeiro, six-time Ultraman champion, who, at 47 years of age, had accumulated 50,000 hours of aerobic training, in a typical dose of 250 METs-h/week. After extensive cardiologic, functional and image evaluation, the only finding worth commenting on was a discreet increase in the left atrium in the MRI.^19^

On the other hand, there are data from observational studies suggesting that there seems to be an increased risk of developing atrial fibrillation,^20^ of having coronary artery calcification, and presenting late enhancement or other abnormalities in cardiac imaging exams.^21^ However, to our best current knowledge, the clinical significance of these "abnormal" findings is still unknown.^18^ In this context, further studies involving large samples of ultra-runners and/or individuals who have been exercisers for over 40 years may contribute to a better understanding of the real cause-effect relation between exercise dose and benefit or harm and/or unfavorable cardiac outcomes.^21^ The fact is that it is currently known that very low doses of aerobic exercise bring benefits, while the maximum limit of the therapeutic dose of aerobic exercise is still unknown. That is, from a medical perspective, there is no evidence that founds a hypothesis of exercise exaggeration.^22^

## Contextualizing in terms of public healthcare

Recent global data suggest that around 5 million deaths per year (almost 10% of total non-violent deaths) are caused by sedentary lifestyles. Moreover, it is estimated that annual worldwide costs due to low levels of physical activity/exercise surpass 67 billion dollars.^23^ In Brazil, government data indicate that tens of millions of Brazilians - almost half the adult population - do not exercise enough. At another extreme, it is estimated that, including athletes and exercisers, much less than 10,000 individuals do intense aerobic training regularly and for periods longer than 10 hours per week or 100 METs-h/week. That is, for every one "extreme" exerciser, there are approximately 5,000 to 10,000 sedentary individuals. It is then clear that the question of a rare and unlikely theoretical excess of exercise for the heart is not a priority in terms of public healthcare.

Therefore, a priority for cardiologists must be the reduction or elimination of the sedentary lifestyle and stimulation of an increase in the exercise dose of the population in general, thus obtaining an improvement in the aerobic condition, and, consequently, a higher life expectancy with the additional bonus of a wide range of beneficial physiological effects. On the other hand, in the case of those rare "extreme" exercisers, we can suggest that they be monitored by qualified professionals when dealing with health and training issues pertinent to this performance profile, while new evidence is gathered on the results of this "extreme" dose of aerobic exercise and its effect on the heart through the future analysis of a cohort made up of 1,200 ultra-runners.^24^
